# Posterior scleral perforation due to endogenous endophthalmitis in a pregnant with selective IgA deficiency

**DOI:** 10.22336/rjo.2022.19

**Published:** 2022

**Authors:** Marina Aguilar-González, Emma Marín-Payá, Marta Pérez-López, Maria Ángeles Bort-Martínez, Juan Aviñó-Martínez, Enrique España-Gregori

**Affiliations:** * Department of Ophthalmology, Hospital Universitario y Politécnico La Fe, Valencia, Spain; ** Department of Oculoplastic and Orbital Surgery, Hospital Universitario y Politécnico La Fe, Valencia, Spain; *** Department of Neuroophthalmology, Hospital Universitario y Politécnico La Fe, Valencia, Spain

**Keywords:** endophthalmitis, endogenous endophthalmitis, IgA deficiency

## Abstract

We present the case of a 35-year-old female patient, pregnant in her third trimester, with no ophthalmologic history of interest and a medical history of IgA deficiency syndrome with bronchiectasis as the only symptomatology, who came to another center with clinical symptoms of ocular discomfort. She was initially diagnosed with anterior uveitis and treated with topical and periocular corticosteroids. Edema and palpebral erythema appeared a few days later and she was diagnosed with idiopathic orbital inflammation and was treated with intravenous (I.V.) corticosteroids, which led to the appearance of a purulent palpebral and subconjunctival collection with a diagnosis of orbital cellulitis. At this time, she came to our center, where ultrasound and magnetic resonance imaging (MRI) showed intraocular and scleral destructuring with scleral perforation. The subconjunctival abscess was drained, being positive for *pseudomonas aeruginosa*, and sputum culture was positive for *Pseudomonas aeruginosa*, so she was diagnosed with endogenous endophthalmitis due to transient *Pseudomonas aeruginosa* bacteremia in the context of IgA deficiency syndrome and treated with antibiotherapy. Despite the improvement of the infectious clinic, the persistence of positive cultures for *pseudomonas aeruginosa* and the evolution to phthisis bulbi at 2 months led to definitive treatment with evisceration. To our knowledge, this is the first reported case of endogenous endophthalmitis associated with IgA deficiency and the first reported case of endogenous bacterial endophthalmitis caused by *pseudomonas aeruginosa* during pregnancy.

## Introduction

The term endophthalmitis encompasses any inflammation within the eye, although in practice it usually refers to those caused by bacterial or fungal intraocular infection [**1**]. Endophthalmitis can be: 1) exogenous, when the microorganism is directly inoculated into the eye after surgery or penetrating trauma; 2) endogenous, when the microorganisms reach the eye via the bloodstream by crossing the blood-retinal barrier [**1**].

Endogenous bacterial endophthalmitis comprises 2-8% [**1**] of endophthalmitis cases. Predisposing conditions, usually immunodeficiency, are present in 60% of them, being the most frequent diabetes mellitus, drug use and malignancy [**1**]. Other predisposing conditions described are renal failure, pneumonia, or rheumatic disease [**1**]. Gram-negative infections are more frequent than gram-positive infections, with *K. pneumonia*, *Pseudomonas aeruginosa* and *N. meningitides* being the most frequently isolated gram-negatives [**1**]. However, the causative organism is not found in all cases [**1**]. 

## Case report

A 35-year-old woman, in third trimester of pregnancy, consulted another center due to right eye (RE) discomfort, being diagnosed with anterior uveitis and treated with topical corticosteroids and subtenon injection of triamcinolone. She had no ophthalmologic history of interest and as medical history she presented IgA deficiency syndrome with bronchiectasis due to repeated respiratory tract infections. Due to the lack of improvement and the development of signs of orbital inflammation (palpebral edema and erythema), she was diagnosed with idiopathic orbital inflammatory disease and she was admitted to hospital and treated with I.V. corticosteroid. During the admission, due to the appearance of palpebral and subconjunctival purulent collection, she was finally diagnosed with orbital cellulitis and was initially treated with ciprofloxacin, which was later changed to cefditoren I.V., with discrete improvement of the symptoms. No imaging tests were requested. For this reason, 1 month after the onset of the whole process, she came to our center looking for a second opinion.

**Fig. 1:**
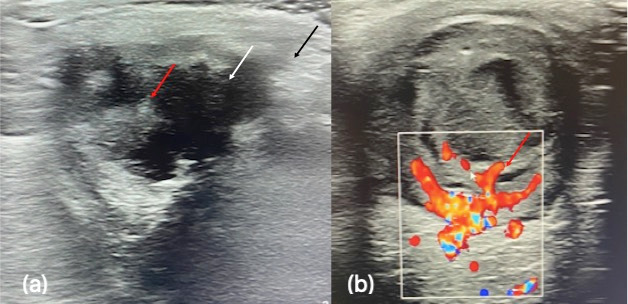
Orbital ultrasound. (**a**) Showed the alteration of the morphology of the eyeball with thickening and alteration of the echogenicity of the covers. It also showed occupation of the vitreous chamber by heterogeneous hyperechogenic material (*red arrow*) and solution of continuity of the temporal ocular wall (*white arrow*) with outflow of the contents and adjacent collection (*black arrow*). (**b**) Doppler study showed destructuring of the posterior wall with vascularized intraocular membranes in relation to probable retinal and choroidal detachment (*red arrow*).

**Fig. 2:**
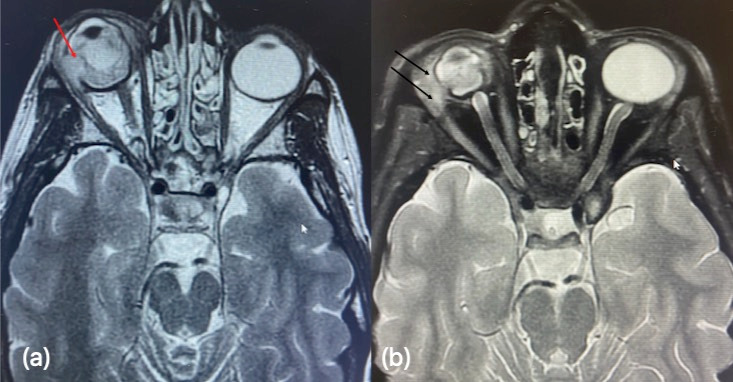
MRI. (**a**) On the first day of admission, the MRI showed an intraocular inflammatory process affecting mainly the posterior compartment with abundant detritus and retinal and choroidal destructuring. It also showed perforation of the temporal sclera with a parietal defect of 8 mm of maximum anteroposterior axis (red arrow) and discrete intraconal extension of the infectious process with slight edematization of retroocular fat and anterior fibers of the external rectus. Finally, intraorbital periocular purulent tissue was observed extending to the internal canthus. (**b**) A month and a half after admission, MRI showed collapse of the right eyeball with concentric thickening of its coverings without purulent signal. On the inferolateral border of the orbit, in an extraconal situation, between the internal rectum and lateral rectum, there were two collections of abscess semiology (*black arrows*)

In our center, physical examination showed abscessed orbital cellulitis, with nasal subconjunctival purulent collection that we drained to obtain samples for culture. The best corrected visual acuity in the RE was amaurosis and intraocular pressure was 5 mmHG. On slit lamp examination, she presented ciliary injection, Tyndall +++, posterior iris synechiae and posterior uveitis with white fundus reflex. The patient was readmitted in our hospital and an ultrasound scan (**Fig. 1(a), (b)**) and an orbital MRI (**Fig. 2(a)**) were requested, showing intraocular and scleral destructuring with posterior scleral perforation (dehiscence of the sclera at the temporal level through which the purulent collection came out). The paranasal sinuses did not show occupation, so sinusitis was discarded as a cause of orbital cellulitis. IgA levels were undetectable in the blood test. 

Numerous sputum cultures had been positive for *Pseudomonas aeruginosa* previous years so sputum samples were taken in order to find infectious focus. The microbiological result of the sputum culture was positive for *Pseudomonas aeruginosa* mucoid phenotype sensitive to amikacin, colistin, gentamicin, meropenem, tobramycin and high doses of ceftazidime demonstrating colonization of the respiratory tract. In view of these findings, endogenous endophthalmitis was established as the most probable diagnosis due to transient *Pseudomonas aeruginosa* bacteremia in the context of IgA deficiency syndrome.

While awaiting the microbiological result, the patient was treated with linezolid and ceftazidime I.V. and topical reinforced vancomycin and ceftazidime every hour and maxidex every 12 hours. Intravitreal injection of antibiotics was discarded due to the existence of ocular perforation.

No microorganisms were isolated in the blood cultures but the result of the abscess samples was positive for *pseudomonas aeruginosa* mucosal phenotype sensitive to amikacin, colistin, gentamicin, tobramycin, meropenem and high doses of ceftazidime, so the same treatment was continued (the use of other antibiotics such as aminoglycosides were ruled out due to pregnancy) and the diagnosis was confirmed.

Linezolid was discontinued after 7 days because no gram-positive bacteria had been detected during the process and treatment with ceftazidime was continued. 

The indication for surgery and evisceration was explained to the patient, but she refused evisceration and opted for conservative antibiotic treatment. Initially, there was an improvement in the periocular inflammation and disappearance of the subconjunctival purulent collection; however, the hypotonia of the eye increased, the cultures continued being positive for *pseudomonas aeruginosa* and there was no improvement in the MRI 2 months (**Fig. 2(b)**) after admission to our center, which showed phthisis bulbi of the RE. During the process, *pseudomonas aeruginosa* was isolated in multiple samples and the microorganism become resistant to ceftazidime and we started treatment with meropenem. From the beginning, the poor prognosis was explained to the patient, and although she initially decided on conservative treatment, given the evolution, after 2 months of conservative treatment she accepted evisceration. The evisceration surgery was uneventful and the samples sent to microbiology were again positive for *pseudomonas aeruginosa*. After surgery, the condition of the orbit and the rest of the eyeball was good and postoperative treatment consisted of meropenem 1g/8h, omeprazole/24 hours and methylprednisolone 40 mg/day.

## Discussion

Although IgA deficiency syndrome usually presents asymptomatically or with upper respiratory tract infections since infancy, which can lead to the development of bronchiectasis, some cases of infection by *pseudomonas aeruginosa* (pneumonia [**2**], malignant otitis externa in neonates [**3**]) have been reported in literature, as in our case, as well as recurrent purulent collections in other parts of the body due to other microorganisms (perianal abscesses, hydrosadenitis and boils in extremities, inguinal abscesses due to *Staphylococcus aureus*) and one case of unilateral palpebral abscess [**4**], with no reported cases of intraocular infections.

Endogenous endophthalmitis during pregnancy, peripartum and postpartum is a rare event with few cases described in literature [**5**]. In most of the reported cases, the isolated microorganism is Candida albicans, while there are only two published cases of bacterial origin (Klebsiella pneumoniae and Sphingomonas paucimobilis) and two in which the responsible germ was not found in vitreous, blood or urine [**5**]. Predisposing factors in pregnant women for the development of endogenous endophthalmitis include intravenous infusion of dextrose or saline during the seventh and eighth month of pregnancy respectively, abortion, appendicitis during pregnancy and premature rupture of membranes [**5**]. However, cases in pregnant women with no other risk factors have also been reported [**5**], so that, pregnancy could be considered a risk factor itself. In our case, two risk factors (pregnancy and IgA deficiency) coexisted.

On the other hand, systemic treatment of bacterial endophthalmitis in pregnant women is a challenge due to the potential risks of the drugs for the fetus [**5**]. Systemic antibiotics such as penicillins, cephalosporins and most erythromycins are considered safe for the fetus; others such as aminoglycosides, among others, may be toxic or teratogenic and should be administered only if there are no other alternatives; finally, there is little evidence on the safety of the use of other antibiotics such as polymyxins and carbapenems [**5**]. This is the reason why our patient was initially treated with high doses of cephalosporins until the germ became resistant and forced treatment with meropenem (we avoided the use of other drugs to which the germ was sensitive but which had potential toxic/teratogenic effects, such as aminoglycosides). 

25% of the cases of endogenous endophthalmitis are initially misdiagnosed [**1**], as it happened in this case. The ophthalmologist should be aware of the signs and symptoms of this disease, because when there is no evident route of entry of the microorganism, a high index of suspicion of endogenous endophthalmitis is required for its early diagnosis and treatment. This is important because late treatment worsens the already poor visual prognosis of this disease [**1**]. 

## Conclusion

We can conclude that *Pseudomonas aeruginosa* is a microorganism that has been described, on the one hand, among the most frequent microorganisms causing endogenous endophthalmitis, and on the other hand, in some cases, as an opportunistic pathogen in patients with IgA deficiency. This case highlights the importance of suspecting endogenous endophthalmitis in any case of unaffiliated ocular inflammation with no obvious route of entry that does not respond favorably to the usual treatments, especially in patients with predisposing conditions, such as pregnancy or immunodeficiency (IgA deficiency syndrome). To our knowledge, this is the first reported case of endogenous endophthalmitis associated with IgA deficiency and the first reported case of endogenous bacterial endophthalmitis caused by *pseudomonas aeruginosa* during pregnancy.

**Conflict of Interest statement**

Authors state no conflict of interest.

**Informed Consent and Human and Animal Rights statement**

Informed consent has been obtained from all individuals included in this study. Patient consent to publish these images was gathered and attached.

**Authorization for the use of human subjects**

Ethical approval: The research related to human use complies with all the relevant national regulations, institutional policies, is in accordance with the tenets of the Helsinki Declaration, and has been approved by the review board of Hospital Universitario y Politécnico La Fe, Valencia, Spain.

**Acknowledgements**

None.

**Sources of Funding**

Authors declare no funding.

**Disclosures**

None.
